# Editorial for the Special Issue on Wide Bandgap Based Devices: Design, Fabrication and Applications

**DOI:** 10.3390/mi12010083

**Published:** 2021-01-15

**Authors:** Farid Medjdoub

**Affiliations:** IEMN (Institute of Electronics, Microelectronics and Nanotechnology), CNRS (Centre National de Recherche Scientifique), Avenue Poincaré, 59650 Villeneuve d’Ascq, France; farid.medjdoub@univ-lille.fr

Emerging wide bandgap (WBG) semiconductors hold the potential to advance the global industry in the same way that, more than 50 years ago, the invention of the silicon (Si) chip enabled the modern computer era. SiC- and GaN-based devices are starting to become more commercially available. Smaller, faster and more efficient than counterpart Si-based components, these WBG devices also offer a greater expected reliability in tougher operating conditions. Furthermore, in this frame, a new class of microelectronic-grade semiconducting materials that have an even larger bandgap than the previously established wide-bandgap semiconductors, such as GaN and SiC, have been created, and are; thus, referred to as “ultra-wide-bandgap” materials. These materials, which include AlGaN, AlN, diamond and BN oxide-based, offer theoretically superior properties, including a higher critical breakdown field, higher temperature operation and potentially higher radiation tolerance. These attributes, in turn, make it possible to use revolutionary new devices for extreme environments, such as high-efficiency power transistors, because of the improved Baliga Figure of Merit, ultra-high voltage pulsed power switches, high efficiency UV-LEDs, laser diodes and RF electronics.

There are 20 papers published in this Special Issue focusing on Wide Bandgap-Based Devices: Design, Fabrication and Applications. Three papers [1,2,3] deal with RF power electronics for future 5G applications and other high-speed high-power applications. Nine of the papers, [4,5,6,7,8,9,10,11,12], explore various designs of wide bandgap high power devices. The remaining papers cover various applications based on wide bandgaps, such as ZnO Nanorods for High Photon Extraction Efficiency of GaN-Based Photonic Emitter [13], InGaZnO Thin-Film Transistors [14], Wide Band Gap WO_3_ Thin Film [15], Silver Nanorings [16,17] and InGaN Laser Diode [18,19,20].

In particular, on RF GaN devices, Kuchta et al. [1] proposed a GaN-based power amplifier design with a reduced level of transmittance distortions. Lee et al. [2] demonstrated a compact 20 W GaN internally matched power amplifier for 2.5 to 6 GHz jammer systems that uses a high dielectric constant substrate, single-layer capacitors, and shunt/series resistors for low-Q matching and low-frequency stabilization. Lin et al. [3] showed a high output power density of 8.2 W/mm in the Ka band by integrating a thick copper metallization.

Concerning GaN power devices, Wu et al. [4] investigated a double AlGaN barrier design toward enhancement-mode characteristics. Ma et al. [5] presented a digitally controlled 2 kVA three-phase shunt APF system using GaN. Tajalli et al. [6] studied the origin of vertical leakage and breakdown in GaN-on-Si epitaxial structures by carrying out a buffer decomposition. The contribution of each buffer layer related to vertical leakage and breakdown voltage could be identified. Sun et al. [7] proposes a new approach to realize normally-off GaN HEMTs using TCAD. The concept is based on the transposition of the gate channel orientation from a long horizontal one to a short vertical one. Mao et al. [8] introduced a portion of the p-polySi/p-SiC heterojunction on the collector side of an IGBT to reduce the turn-off loss without sacrificing other characteristics of the device. Kim et al. [9] implemented a SiC micro-heater chip as a novel thermal evaluation device for next-generation power modules and to evaluate the heat resistant performance. Keum et al. [10] investigated the time-dependent dielectric breakdown (TDDB) characteristics of normally-off AlGaN/GaN gate-recessed MISHEMTs submitted to proton irradiation. Abid et al. [11] presented the fabrication of AlN-based thin and thick channel AlGaN/GaN heterostructures that have been regrown by molecular beam epitaxy on AlN/sapphire. A remarkable breakdown field of 5 MV/cm has been observed for short contact distances, which is far beyond the theoretical limit of the GaN-based material system. Sandupatla et al. [12] used vertical GaN-on-GaN Schottky diodes as α- particle radiation sensors. They reported the highest reverse breakdown voltage of −2400 V from Schottky barrier diodes on a freestanding GaN substrate with 30 μm drift layer.

Besides, Lee et al. [13] demonstrated self-aligned hierarchical ZnO nanorod nanosheet arrays on a conventional photonic emitter with a wavelength of 430 nm with an improved optical output power. Zhang et al. [14] improved the electrical performance and bias-stress stability of amorphous InGaZnO thin-film transistors using buried-channel devices with multiple-stacked channel layers. Liu et al. [15] developed a tungsten trioxide (WO3) wide band gap using ammonium tungstate to obtain a high electrochromic modulation ability roughly 40% at 700 nm wavelength. Li et al. [16] optimized silver nanoring for transparent flexible electrodes applied to wide bandgap devices. Y. Wang et al. [17] proposed, for the first time, a novel GaN-based heterostructure Gunn diode, which turns out to be an excellent solid-state source for terahertz oscillators. W. Wang et al. [18] carried out a theoretical investigation the optical field distribution and electrical property improvements of the InGaN laser diode with an emission wavelength around 416 nm. Device optimization is favorable for the achievement of low threshold current and high output power lasers. Deng et al. [19] describes an optimization of InGaN/GaN distributed feedback laser diodes to enhance the efficiency. Finally, Luo et al. [20] propose a design based on a p-type composition-graded Al_x_Ga_1−x_N electron blocking layer to improve the output power of GaN-based VCSEL.

I would like to take this opportunity to thank all the authors for submitting their papers to this Special Issue. I would also like to thank all the reviewers for dedicating their time and helping to improve the quality of the submitted papers.
